# Use of Metformin Alone Is Not Associated with Survival Outcomes of Colorectal Cancer Cell but AMPK Activator AICAR Sensitizes Anticancer Effect of 5-Fluorouracil through AMPK Activation

**DOI:** 10.1371/journal.pone.0097781

**Published:** 2014-05-21

**Authors:** Xinbing Sui, Yinghua Xu, Jie Yang, Yong Fang, Haizhou Lou, Weidong Han, Maolin Zhang, Wei Chen, Kaifeng Wang, Da Li, Wei Jin, Fang Lou, Yu Zheng, Hong Hu, Liu Gong, Xiaoyun Zhou, Qin Pan, Hongming Pan, Xian Wang, Chao He

**Affiliations:** 1 Department of Medical Oncology, Sir Run Run Shaw Hospital, Zhejiang University, Hangzhou, China; 2 Biomedical Research Center and Key Laboratory of Biotherapy of Zhejiang Province, Hangzhou, China; 3 Department of Colorectal Surgery, Sir Run Run Shaw Hospital, Zhejiang University, Hangzhou, China; Massachusetts Eye & Ear Infirmary, Harvard Medical School, United States of America

## Abstract

Colorectal cancer (CRC) is still the third most common cancer and the second most common causes of cancer-related death around the world. Metformin, a biguanide, which is widely used for treating diabetes mellitus, has recently been shown to have a suppressive effect on CRC risk and mortality, but not all laboratory studies suggest that metformin has antineoplastic activity. Here, we investigated the effect of metformin and AMPK activator AICAR on CRC cells proliferation. As a result, metformin did not inhibit cell proliferation or induce apoptosis for CRC cell lines in vitro and in vivo. Different from metformin, AICAR emerged antitumor activity and sensitized anticancer effect of 5-FU on CRC cells in vitro and in vivo. In further analysis, we show that AMPK activation may be a key molecular mechanism for the additive effect of AICAR. Taken together, our results suggest that metformin has not antineoplastic activity for CRC cells as a single agent but AMPK activator AICAR can induce apoptosis and enhance the cytotoxic effect of 5-FU through AMPK activation.

## Introduction

Colorectal cancer (CRC) is still a leading cause of cancer-related morbidity and mortality around the world, although a lot of progress has been made in the treatment of CRC over the past years [1], [2]. Epidemiologic studies have shown that diabetes mellitus (DM) increases incidence and mortality of cancers, especially gastrointestinal malignancy [3], [4]. There is increasing evidence linking diabetes mellitus with an increased risk of colorectal cancer [5], [6]. However, some other studies have not supported this view. A multi-center, double-blind, placebo-controlled, randomized controlled trial showed that there was no statistically significant difference in colon-cancer specific survival in those who with diabetes [7]. So, the relationship between DM and CRC risk remains controversial.

Metformin (1,1-dimethylbiguanide hydrochloride), a biguanide derivative which is widely used for treating diabetes mellitus, has been shown to exert potentially important anticancer effects [8], [9], but others have not supported this view [10], [11]. The mechanisms involved in the antineoplastic effects of metformin are probably very diverse, including activation of adenosine monophosphate kinase (AMPK) [12], phosphatidylinositol-3 kinase (PI3K) mutation [13], p53 deficiency [14] and so on. Among these mechanisms, the AMPK- mammalian target of rapamycin (mTOR) axis plays a central role for the antineoplastic effects of metformin. Both metformin and 5-amino-imidazol-4-carboxamide-1-b-4-ribofuranoside (AICAR) can activate AMPK pathway. AMPK is a serine/threonine kinase and a cellular fuel sensor pathway sensitive to the increase of the AMP/ATP ratio, which has been connected to several human tumor suppressors [15]. The effects of metformin are mainly explained by the activation of AMPK, which inhibits protein synthesis and gluconeogenesis during cellular stress [16].

So far 5-Fluorouracil (5-FU) remains a widely used chemotherapeutic drug in the treatment of colorectal carcinoma. Recently, metformin is reported to have a synergistic effect in combination with some chemotherapeutic agents [17], [18]. However, it remains unclear whether metformin or AICAR can be used in combination with 5-FU to enhance the anticancer effect, since there is no study on the correlation between the metformin/AICAR and 5-FU treatment in vitro and in vivo.

We investigated the impact of metformin and AMPK activator AICAR on CRC cell proliferation. Here we demonstrate that use of metformin alone is not associated with survival outcomes of colorectal cancer cell but AICAR can induce apoptosis and enhance the cytotoxic effect of 5-FU through AMPK activation, which should be considered in the ongoing clinical trials where metformin are used in the treatment of colorectal cancer.

## Results

### Metformin did not Inhibit Colorectal Cancer Cell Growth

**Figure 1:**
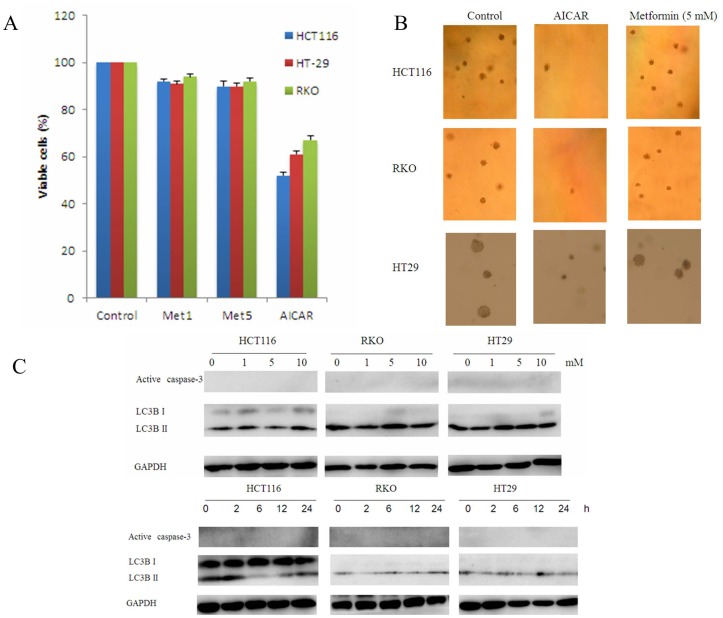
Metformin did not inhibit colorectal cancer cell growth and induce apoptosis or autophagy. (A) HCT116, RKO and HT29 were seeded in 96-well plates. After 24 h, metformin (1 and 5 mM) and AICAR (5 mM) were added to the culture media. 24 h after the addition of the agents, the effect of metformin on colorectal cancer cell survival was performed a cell viability assay (MTT). (B) Photographs of soft agar colonies of HCT116, RKO and HT29 cells 2 weeks after the treatment with 5 mM metformin and 5 mM AICAR. (3) Cancer cell lines were treated with different concentrations of metformin (1, 5 and 10 mM) for various time, then, the expression of active caspase-3 and LC3-II/I ratio was assessed by western blotting.

In order to examine whether metformin affects human colorectal cancer cell proliferation we investigated the effect of the drug on three cancer cell lines: HCT116, RKO and HT29 cells. Cells were grown in 10% fetal bovine serum (FBS), treated with metformin (1 and 5 mM) and AICAR (5 mM) as a control. AICAR is known to induce apoptosis. The MTT viability assay was performed after the addition of the agents for 24 h. As a result, AICAR reduced cell viability by 50–70% in the three cell lines, but little decrease of cell viability was found in the three cell lines treated with metformin (Figure 1A), indicating metformin might have no effect on colorectal cancer cell growth. To determine whether metformin inhibits anchorage-independent growth, we performed a soft-agar colony formation assay in absence or presence of 5 mM metformin renewed daily. After 2 weeks, the cells were counted under a microscope. In agreement with MTT viability assay results, metformin did not decrease the number and the size of the colonies (Figure 1B). These results suggest that metformin did not possess growth inhibitory activity in colorectal cancer cell.

### Metformin did not Induce Apoptosis, Autophagy and Cell Cycle Arrest

**Figure 2:**
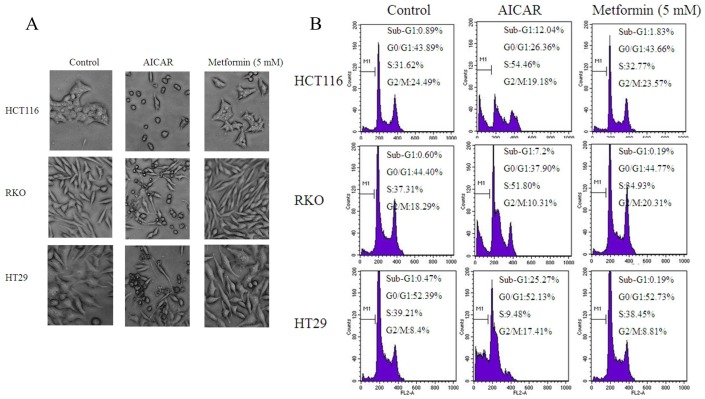
Metformin did not induce apoptosis and cell cycle arrest. (A) 24 h after the addition of 5 mM metformin and 5 mM AICAR, the effect of metformin on colorectal cancer cell survival was observed. Representative cell morphological changes detected by light microscopy; characteristic morphological features of apoptosis were observed, including detachment and cell shrinkage. (B) Fractions of cells in the sub-G1, G0/G1, S or G2/M phases of the cell cycle are investigated after the treatment with metformin and AICAR.

To investigate whether metformin induce apoptosis and autophagy, three cancer cell lines were treated with different concentrations of metformin (1, 5 and 10 mM) for various time, then, the expression of active caspase-3 and LC3-II/I ratio was assessed by western blotting. As shown in Figure 1C, apoptosis and autophagy were not activated in dose- and time-dependent manner when the cells were treated with metformin. For further confirmation, all these treated cells were analyzed by electron microscopy and flow cytometry. These three cells displayed extensive apoptotic cells (Figure 2A) and an increased sub-G1 population (Figure 2B) after AICAR treatment. In contrast, very few apoptosis were seen in metformin treated cells. We then asked whether metformin affects cell cycle. As seen in Figure 2B, we observed the same percentage of cells in G0/G1, G2and S phase in metformin treated cells compared to their controls. Taken together, our data demonstrate that metformin did not induce apoptosis, autophagy and cell cycle arrest.

### AICAR Potentiated Anti-cancer Effect of 5-FU in vitro and in vivo

**Figure 3:**
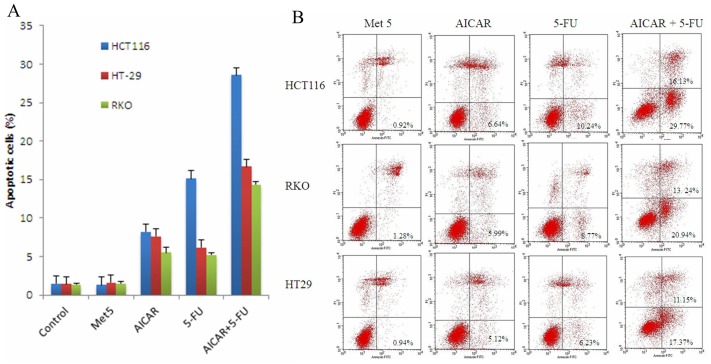
AICAR potentiated anti-cancer effect of 5-FU in vitro. (A) HCT116, RKO and HT29 were seeded in 96-well plates. After 24 h, metformin (5 mM), AICAR (5 mM), 5-FU (20 µM) and AICAR plus 5-FU were added to the culture media. 24 h after the addition of the agents, the effect of metformin and AICAR on colorectal cancer cell survival was performed a cell viability assay (MTT). (B) Representative results of annexin V-FITC/PI staining and quantitative analysis; values are mean ± SD of three independent experiments; *p<0.05.

It has been acknowledged that 5-FU is usually used in combination with other chemotherapeutic drugs to enhance its therapeutic efficacy for CRC patients. In order to examine whether AICAR can sensitize anticancer effect of 5-FU, we evaluated the effect of AICAR on 5-FU-induced apoptosis. We found that the viability of cells treated by AICAR in combination with 5-FU was significantly lower than that of controls (Figure 3A). Flow cytometry showed that the percentage of apoptotic cells was significantly higher in cells treated by AICAR in combination with 5-FU compared to controls (Figure 3B). These results suggest that AICAR enhances 5-FU-induced apoptosis in colorectal cancer cells.

**Figure 4:**
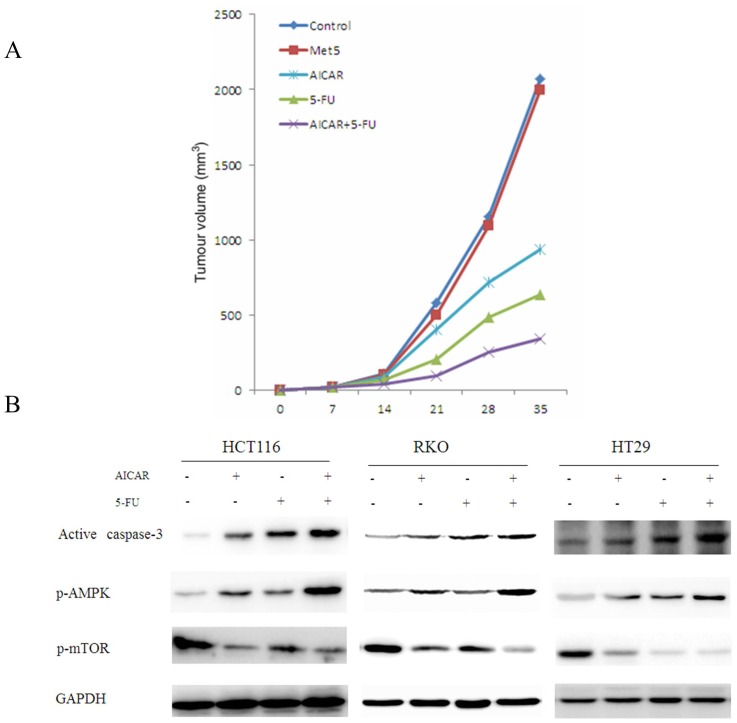
AICAR potentiated anti-cancer effect of 5-FU in vivo and AMPK activation mediated by AICAR. (A) Growth curve of xenograft tumors treated with indicated drugs. (B) The effect of AICAR on AMPK activation. The expression of active caspase-3, p-AMPK and p-mTOR were analyzed by Western blotting.

To examine if metformin and AICAR could affect tumor growth in vivo, we then injected nude mice subcutaneously with HT29 cells. The mice with tumor xenografts reaching 100 mm^3^ were randomly divided in to 5 experimental groups: control group, metformin 200 mg/ml/d (in drinking water, which corresponds to 15 mg/kg) group, AICAR 400 mg/kg/2 days group, 5-FU 40 mg/kg/2 days group and AICAR plus 5-FU group for 5 weeks. Treatment was administered via intratumoral injection. All mice tolerated this treatment well without significant toxicity and had stable body weights. First, metformin levels were assayed (3 h and 15 h after metformin injection) by using high-performance liquid chromatography and the metformin levels in mice sera were on average 1.15 (±0.31) µg/ml for peak which equaled about human peak levels for 500 mg p.o. (orally) daily. The experiments were performed in triplicate and the result showed that the levels of metformin in the plasma of female nude mice were usually achieved in the humans. Then the tumor sizes from the xenografts were measured. As a result, tumor size from the xenografts of AICAR group not metformin group was smaller compared to control group. Moreover, the sizes of AICAR plus 5-FU group were significantly smaller compared to AICAR alone or 5-FU group, suggesting that AICAR could potentiate 5-FU-induced anticancer effect of HT29 (Figure 4A).

### AICAR Might Enhance Anticancer of 5-FU through AMPK Activation

It has been acknowledged that the effects of AICAR are mainly explained by the activation of AMPK, which regulates cellular energy metabolism. To investigate whether AMPK activation might be associated with the additive effect of AICAR, phosphorylation of AMPK and mTOR were assessed by western blotting using specific antibodies. We found that p-AMPK induced by 5-FU were enhanced by AICAR, together with decreased p-mTOR and increased active caspase-3 (Figure 4B), indicating that activation of AMPK by AICAR potentiated 5-FU-induced apoptosis.

## Discussion

Although increasing evidence support the idea that hyperinsulinemia and hyperglycemia promote carcinogenesis in patients with diabetes mellitus [19], [20], it remains controversial whether the risk of colorectal cancer is also associated with diabetes mellitus and metformin treatment might reduce risk of CRC [10].

Metformin, a biguanide derivative, is an extensively used and well-tolerated drug for treatment of type 2 diabetes mellitus. The effectiveness of metformin as an antidiabetic drug is explained by its ability to decrease hepatic gluconeogenesis and stimulate glucose uptake in muscle, resulting in reduced circulating glucose concentrations [21]. Metformin levels in humans are in micro molar levels but gut may or may not see mili-molar levels. Recent data suggest that metformin could protect from cancer including CRC and has anti-tumor effects in mouse xenografts [22], [23]. Interestingly, there is a conflicting report regarding the potential role of metformin and cancer. Bodmer and colleagues found that use of metformin was not associated with a decreased risk of colorectal cancer and metformin also did not alter the risk of lung cancer [10], [24]. It is also reported that there was no statistically significant association between metformin exposure and disseminated colorectal cancer at diagnosis [25]. Metfromin may not work on regular tumor cells but work on stem cells. It has been shown that metformin could selectively target cancer stem cells [26], and act together with chemotherapy to block tumor growth and prolong remission in multiple cancer cell types [26], [27]. Metformin can also inhibit the inflammatory response associated with cellular transformation and cancer stem cell growth [28]. In addition, metformin can accelerate the growth of BRAF V600E-driven melanoma by upregulating VEGF-A [29] and promote the angiogenic phenotype in the ERalpha negative MDA-MB-435 breast cancer model [30]. Overall, metformin may only have effects in preventing tumor initiation but after the cancer has been established it may not have an effect. Our data also showed that metformin exposure did not inhibit colorectal cancer cell growth, induce apoptosis or autophagy and cell cycle arrest. In agreement with in vitro, in vivo study revealed that metformin did not suppress tumor growth but AMPK activator AICAR emerged antitumor activity. Therefore, metformin might have no antineoplastic activity for CRC cells as a single agent.

The anticancer effects of AICAR are mediated by the activation of AMPK and reduction of mTOR signaling [31]. AMPK activation can suppress mTOR pathway to inhibit cell growth and proliferation. AICAR have been reported to enhance the efficacy of conventional chemotherapeutic agents in the treatment of local and metastatic nasopharyngeal carcinoma (NPC) [32]. AMPK activators such as AICAR provide a therapeutic strategy for hematological malignancies [33], [34]. First, AICAR can induce apoptosis in B-cell chronic lymphocytic leukemia cells [35] and kill chronic myelogenous leukemia (CML) cells through PKC-dependent induction of autophagic cell death [36]. Second, AICAR has antileukemic effects on BCR-ABL-expressing cells [37] and childhood acute lymphoblastic leukemia (ALL) cells [38]. Third, AICAR can induce G(1)/S arrest and Nanog downregulation via p53 and enhance erythroid differentiation [39]. Finally, AICAR can also induce apoptosis independently of AMPK and p53 through up-regulation of the BH3-only proteins BIM and NOXA in chronic lymphocytic leukemia cells [40]. In addition to NPC and leukemia, AICAR is involved in neural stem cell growth suppression and cell cycle arrest by down-regulating phospho-retinoblastoma protein and cyclin D [41]. AICAR can inhibit the growth of retinoblastoma by decreasing angiogenesis and inducing apoptosis [42] or activation of AMPK [43]. AICAR is also demonstrated to inhibit the proliferation of EGFRvIII expressing glioblastoma through AMPK pathway [44]. Moreover, AICAR can be used in clinical trials as a cardioprotectant under ATP-depleted conditions and has been shown to be an exercise mimetic in animals [45]. In agreement with these results, we reported that AICAR can induce apoptosis to emerge antineoplastic activity. Furthermore, AICAR enhanced the cytotoxic effect of 5-FU through AMPK activation.

In conclusion, our study revealed that use of metformin alone is not associated with survival outcomes of colorectal cancer cell but AMPK activator AICAR can induce apoptosis and emerge antineoplastic activity. Furthermore, activation of AMPK might be a key cause that AICAR can enhance the cytotoxic effect of 5-FU in colorectal cancer cells.

## Materials and Methods

### Cell Culture

The human colorectal carcinoma cell lines HCT116, RKO and HT29 were purchased from ATCC (LGC Standards SLU, Barcelona, Spain). The cell lines were maintained in McCoy’s 5A or Dulbecco’s modified Eagle’s medium (DMEM; Gibco BRL, Rockville, MD, USA) with 10% fetal bovine serum (FBS), 100 units/mL penicillin, 100 µg/mL streptomycin (Invitrogen), and 2mmol/L L-glutamine at 37°C in a humidified atmosphere of 95% air and 5% CO_2_.

### Materials

5-FU was purchased from Jinyao Amino Acid Co., Ltd. (Tianjin, China). AICAR and metformin were purchased from Sigma-Aldrich. Anti-phospho-eIF2α and phosphor-AMPK were purchased from Cell Signaling.

### Measurement of Cell Viability and Apoptosis

Cell viability was determined by MTT assay. Cells were seeded in 96-well flat bottom microtiter plates at a density of 1×10^4^ cells per well. The agents were added at the concentrations indicated for 24 h. The absorbance was measured on a microplate reader (Synergy HT, Bio-Tek, USA) at 570 nm.

Phartmingen annexin V-FITC Apoptosis Ddtection Kit I (BD, USA) was used to detect apoptosis and the estimation procedure was performed according to the manufacturer’s instructions. 2×10^6^ cells were seeded into a 6 cm dish. After attachment overnight, cells were washed twice with PBS and the medium was replaced medium with 5 mM metformin or 20 µg/ml 5-FU. All cells including the floating cells in the culture medium were harvested. The cells were resuspended in ice-cold 1×binding buffer at a concentration of 1×10^6^ cells/ml. 100 µl of cell suspension were each mixed with 5 µl FITC Annexin V and 5 µl PI. The mixture was incubated for 15 min at room temperature in the dark and then analyzed by FACSCalibur Flow Cytometer (BD Biosystems, Heidelberg, Germany).

### Soft-agar Colony Formation Assay

500 cells were suspended in medium containing 0.3% low-melt agarose, seeded into a six-well plate that was overlaid with 0.5% low-melt agarose, and allowed to grow for 2 weeks at 37°C in 5% CO_2_. The colonies containing more than 50 cells were counted under a microscope. Three wells were analysed for each experiment.

### Cell-cycle Analysis

Cells were trypsinized, washed twice in ice-cold PBS, and resuspended in 200 µl citrate buffer (250 mM sucrose, 40 mM Tri-sodium citrate, 5% dimethyl sulfoxide (DMSO) adjusted to pH 7.6 with 40 mM acetic acid). Propidium iodide (40 µg/ml) solution was added to the cells and incubated for 45 min in the dark at 4°C prior to analysis.

### Western Blot Analysis

Cells were harvested from cultured dishes and were lysed in a lysis buffer [20 mM Tris-HCl pH 7.6, 1 mM EDTA, 140 mM NaCl, 1% NP-40, 1% aprotinin, 1 mM phenylemethylsulfonyl fluoride (PMSF), 1 mM sodium vanadate]. Protein concentration was determined using a BCA Protein Assay Kit (Pierce). Cell lysates (40 µg protein/line) were separated on a 5 to 20% Tris-Tricine Ready Gel SDS-PAGE (Bio-Rad) for nitrocellulose membrane blotting. The blotted membranes were blocked with 5% skim milk for 1 h and were incubated with primary antibodies. The immunoreactive bands were visualized by enhanced chemiluminescence using horseradish perox-idase-conjugated IgG secondary antibodies. Band density was measured by densitometry, quantified using gel plotting macros of NIH image 1.62, and normalized to an indicated sample in the identical membrane.

### In vivo Subcutaneous Tumor Model

All of the in vivo experimental protocols were approved by the animal care committee of Sir Run Run Shaw Hospital, Zhejiang University. Viable HCT116 cells (1×10^7^cells in 0.1ml phosphate buffer saline) were injected subcutaneously into right dorsal flank of 6-week-old female BALB/c nude mice (six mice per group). Tumor volume was assessed every 2 days for 4 weeks. Tumor volume was calculated by the following formula: (short diameter)^2^×(long diameter)/2.

### Statistical Analyses

Results are expressed as values of mean ± standard deviation (SD). Statistical analysis was performed using SPSS 11.0 for Windows (SPSS Inc., Chicago, IL, USA). We performed paired t-test (two-tailed) statistical analysis, statistical significance was set at p<0.05.
